# Languages for different health information readers: multitrait-multimethod content analysis of Cochrane systematic reviews textual summary formats

**DOI:** 10.1186/s12874-019-0716-x

**Published:** 2019-04-05

**Authors:** Jasna Karačić, Pierpaolo Dondio, Ivan Buljan, Darko Hren, Ana Marušić

**Affiliations:** 1 Croatian Association for the Promotion of Patients’ Rights, Split, Croatia; 2 School of Computer Science, Technological University Dublin, Dublin, Ireland; 3 Cochrane Croatia and Department of Research in Biomedicine and Health, University of Split School of Medicine, Šoltanska 2, 21000 Split, Croatia; 4 University of Split Faculty of Humanities and Social Sciences, Split, Croatia

**Keywords:** Consumer health information, Comprehension, Health literacy, Natural language processing

## Abstract

**Background:**

Although subjective expressions and linguistic fluency have been shown as important factors in processing and interpreting textual facts, analyses of these traits in textual health information for different audiences are lacking. We analyzed the readability and linguistic psychological and emotional characteristics of different textual summary formats of Cochrane systematic reviews.

**Methods:**

We performed a multitrait-multimethod cross-sectional study of Press releases available at Cochrane web site (*n* = 162) and corresponding Scientific abstracts (*n* = 158), Cochrane Clinical Answers (*n* = 35) and Plain language summaries in English (*n* = 156), French (*n* = 101), German (*n* = 41) and Croatian (*n* = 156). We used SMOG index to assess text readability of all text formats, and natural language processing tools (IBM Watson Tone Analyzer, Stanford NLP Sentiment Analysis and Linguistic Inquiry and Word Count) to examine the affective states and subjective information in texts of Scientific abstracts, Plain language summaries and Press releases.

**Results:**

All text formats had low readability, with SMOG index ranging from a median of 15.6 (95% confidence interval (CI) 15.3–15.9) for Scientific abstracts to 14.7 (95% CI 14.4–15.0) for Plain language summaries. In all text formats, “Sadness” was the most dominantly perceived emotional tone and the style of writing was perceived as “Analytical” and “Tentative”. At the psychological level, all text formats exhibited the predominant “Openness” tone, and Press releases scored higher on the scales of “Conscientiousness”, “Agreeableness” and “Emotional range”. Press releases had significantly higher scores than Scientific abstracts and Plain language summaries on the dimensions of “Clout”, and “Emotional tone”.

**Conclusions:**

Although the readability of Plain language summaries was higher than that of text formats targeting more expert audiences, the required literacy was much higher than the recommended US 6th grade level. The language of Press releases was generally more engaging than that of Scientific abstracts and Plain language summaries, which are written by the authors of systematic reviews. Preparation of textual summaries about health evidence for different audiences should take into account readers’ subjective experiences to encourage cognitive processing and reaction to the provided information.

**Electronic supplementary material:**

The online version of this article (10.1186/s12874-019-0716-x) contains supplementary material, which is available to authorized users.

## Background

Health literacy is defined as the “constellation of skills, including the ability to perform basic reading and numerical tasks required to function in the health care environment” [1]. It has been recognized for a long time as an important factor both for understanding health information and prediction of health status [1, 2]. Health information should be easily accessible to people with low levels of health literacy [3], which means that written information should be easily readable and written in a plain language [4], preferably a sixth-grade reading level in the USA [5], which translates to the primary education of 11 to 12 year-olds. A number of studies have shown that materials containing information about health are often presented above patients’ readability level [5–8]; also, patients and the general population have low health literacy [9], with resultant deficits in decision-making [10]. The most common readability tool for health information is the SMOG index (“Simple Measure Of Gobbledygook”). It emerged as the most appropriate among 12 different readability formula tested for their characteristics and predictive validity for public and patient material produced by the National Cancer Institute in the US [8]. Studies into other language characteristics also point to the importance of subjective expressions of attitudes, sentiments and feelings, as well as linguistic fluency for processing and interpreting the facts presented in a textual information [11, 12].

While there are many efforts to translate health information of different kinds to a form suitable for patients and the general public [4–8], particular challenge for achieving appropriate health literacy are systematic reviews of health interventions, because they summarize evidence from individual studies to help doctors and patients make informed choices about health treatments. An example of the effort to make customized health information is Cochrane, an international professional community dedicated to producing systematic reviews of health interventions [13]. Cochrane has made a massive effort to develop separate summary presentation formats for different users [13]. The basic form of summarizing the results of a Cochrane systematic review is the *Scientific abstract*. It is written by review authors and aims at health professionals and researchers. Healthcare practitioners and professionals are also targeted with *Cochrane Clinical Answers*, which are “readable, digestible, clinically focused” presentation formats, produced by Cochrane [14]. *Press releases* are summary formats about Cochrane systematic reviews provided for media professionals; these are written by Cochrane. Finally, *Plain language summaries* are formats written for the lay public – consumers, patients and their families, and are translated into several languages. Plain languages summaries are written by review authors, but they often do not follow Cochrane writing standards [15], and are thus diverse in style, words usage, and possibly in literacy requirements.

The aim of our study was to compare the linguistic characteristics of different textual formats about the same health information. We used the availability of different summary formats for individual Cochrane systematic reviews to assess the linguistic characteristics of these formats. We compared their readability and used three different natural language processing tools to assess features that reflect different psychological and emotional processes embedded in different textual summary formats.

## Methods

### Study design and data sources

We used a cross sectional study design and multitrait-multimethod approach to analyze the linguistic characteristics of the set of all 164 Press releases about Cochrane systematic reviews available in February 2016 at http://www.cochrane.org/media, and corresponding Scientific abstracts, Cochrane Clinical Answers and Plain language summaries in English, French, German and Croatian. The textual formats were entered by one author (JK) into an Excel file, and formatted by removing all subheadings to create a single text paragraph. Another author (AM) checked the data entry quality and completeness.

### Text readability

The readability of summary formats in English and translations of plain language summaries in German and French was assessed using the “Simple Measure Of Gobbledygook” (SMOG) index [16]. The interpretation of the SMOG index for health information area is that values over 6 (meaning the level of education at US 6th grade, i.e. 11 to 12 year-olds) are considered difficult for a non-specialist reader. SMOG index for texts in English, French and German was measured using an online program (www.readable.io). The same program was used to calculate the number of words, number of sentences, words per sentence and syllables per word for all summary format samples. The readability of the plain language summaries in Croatian was calculated using the SMOG formula adapted to Croatian [17] and successfully used to analyze medical information for patients in Croatia [18].

### Linguistic characteristics of the text

We used IBM Watson Tone Analyzer [19] to assess the tone and style of the text formats. This software examines the tone of the language in a text on three levels: emotional (e.g., angry, cheerful, negative), personality (e.g., agreeable, conscientious, open), and writing (e.g., analytical, tentative) [19, 20].

The sentiment of the text was assessed using two different programs: Linguistic Inquiry and Word Count (LIWC) [21] and Stanford Natural Language Processing (NLP) Sentiment Analysis Module [22, 23]. LIWC uses sets of words identified as typical of specific psychological processes, based on analyses of large amounts of texts. These word sets describe different entities or processes and can give estimates how much a text uses words that indicate specific process or psychological factors. The Stanford NLP Sentiment Analyzer was implemented using a machine learning approach, training a deep learning model over a tree-representation of each sentence [24]. The Stanford Core NLP Analyzer was also used to extract the predominant sentiment of the text, as well as various stylistic and grammatical features from the text, such as number of adjectives, nouns, presence of named entities mentioned in the text (e.g., organizations, locations, persons), and parts of speech tags [24].

Linguistic characteristics of Cochrane Clinical Answer formats were not analyzed due to a sample size that was too small for a meaningful analysis. Translation of Plain language summaries into German, French and Croatian were also not analyzed because those language corpora are not well presented in linguistic analysis programs.

### Statistical analysis

Word count, number of sentences in the summary format, words per sentence and syllables per words were presented as medians with 95% confidence intervals due to the non normality of distributions tested by the Kolmogorov Smirnov distribution test. The results of tone and sentiment analysis were presented as averages with 95% confidence intervals.

We used the Kruskal Wallis test and *post-hoc* Conover Iman test for group comparison. We also used the Kruskal Wallis test to compare the results for different plain language translations. Comparison of results was conducted using both frequentist statistical techniques and Bayesian hypothesis testing. We compared the results for tone and sentiment analysis using ANOVA and Tukey post hoc test, and partial η-squared coefficient for effect sizes. Bayes Factor (BF_10_) was used as a quantitative expression of relative probability for the alternative hypothesis compared to the null hypothesis. Bayes Factor was calculated using JASP 0.8.3.1 (https://jasp-stats.org/) and assuming a default prior distribution (Cauchi distribution) [25]. Bayes Factors above 3 indicated substantial evidence for the alternative hypothesis [25]. The full set of summary formats is available upon request from the authors.

## Results

**Fig. 1:**
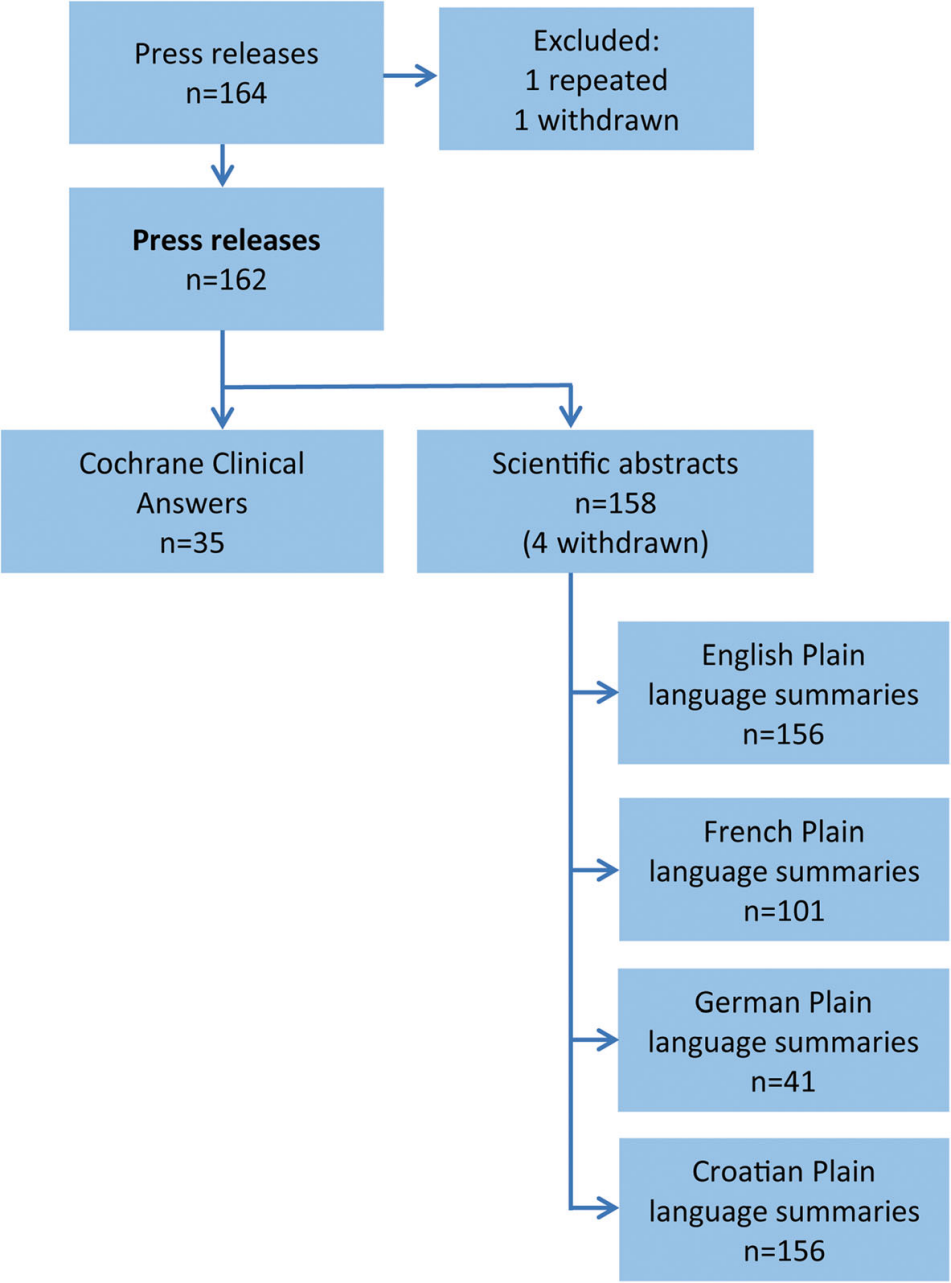
Cochrane systematic review summary text formats included in the analysis. The starting cohort for sample formation was the collection of Cochrane Press releases in February 2016

For 162 unique Press releases at the Cochrane web-site, we could identify 35 Cochrane Clinical Answers, 158 Scientific abstracts, 156 English Plain language summaries and their translations into French (*n* = 101), German (*n* = 41) and Croatian (*n* = 156) (Fig. 1).

### Readability

**Fig. 2:**
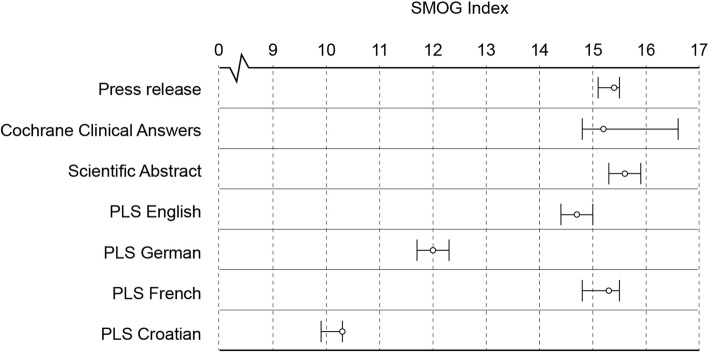
SMOG (“simple measure of gobbledygook”) readability index (number of years of education needed for a person to understand a written text) of Cochrane Scientific abstracts (SA) (*n* = 158); Press releases (PR) (*n* = 162); Plain language summaries (PLS) in English (*n* = 156), German (*n* = 41), French (*n* = 101) and Croatian (*n* = 156); and Cochrane Clinical Answers (CCA) (*n* = 35). Error bars are 95% confidence intervals. The full analysis of readability dataset is available Additional file 1: Table S1 and S2. SMOG index for Croatian language was calculated according to the formula adapted to Croatian [17]. Statistically significant differences (Kruskal Wallis test and post-hoc Conover Iman test): for summary formats in English – PR vs SA and PLS, CCA vs. PLS, SA vs. PR and PLS, PLS vs. all other formats; for PLS translations – all comparisons were significant

The readability of the Plain language summaries in English and their translations to French, German and Croatia was significantly lower than that of the Scientific abstract, Cochrane Clinical Answer and Press release formats, as indicated by their lower vs. higher SMOG scores, respectively (Fig. 2; see the Additional file 1: Table S1 and Table S2 for full data set analysis). For all formats, the SMOG score was over 10, indicating the readability level higher than US 10th-grade reading level.

### Tone analysis

**Fig. 3:**
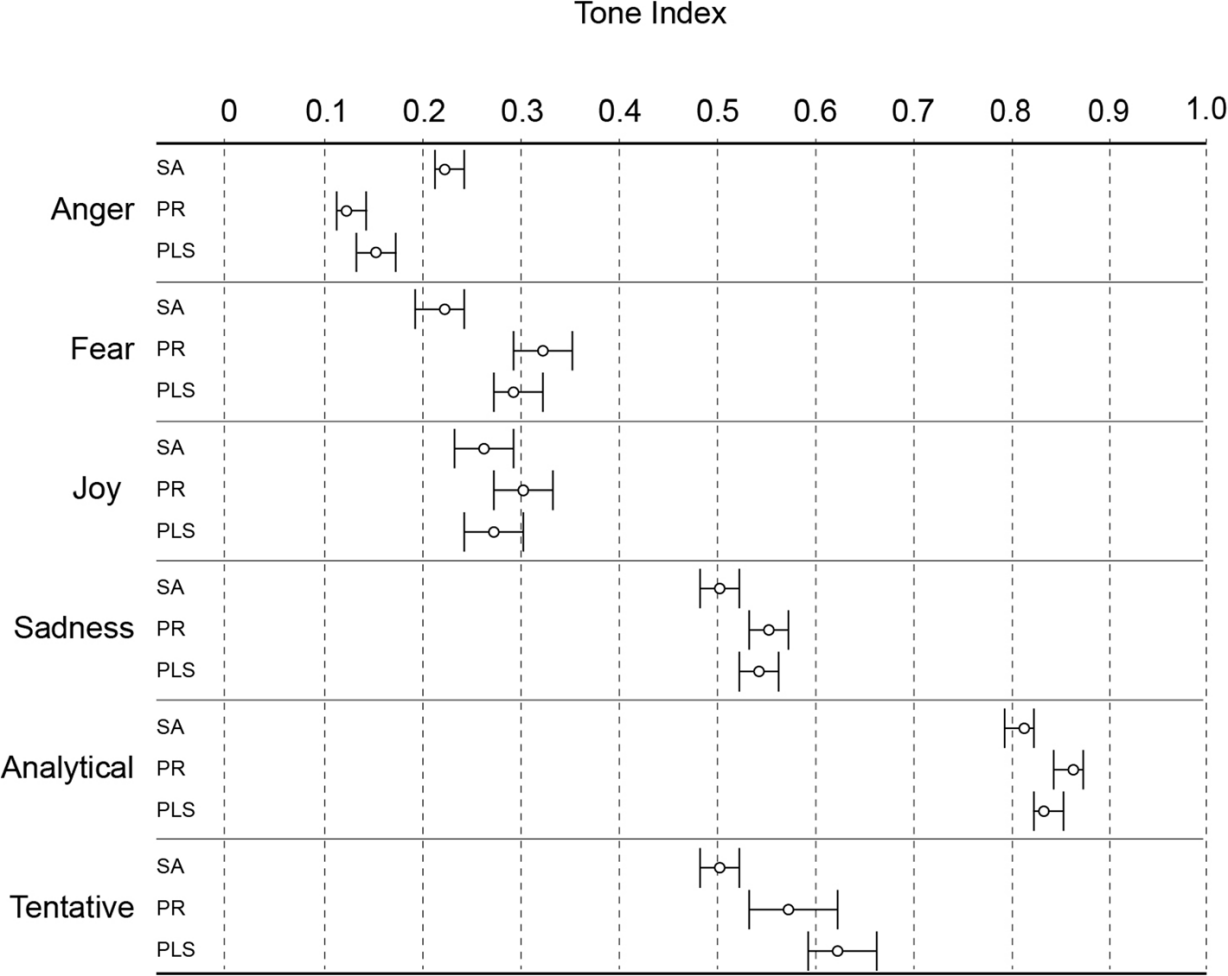
Emotional and writing tone analysis of Cochrane Scientific abstracts (SA) (*n* = 158), Press releases (PR) (*n* = 162), and English Plain language summaries (PLS) (*n* = 156). The results of IBM Watson Tone Analyzer are expressed as the probability of the output variable to be present in the text. Error bars are 95% confidence intervals. The full analysis of the dataset is available in Additional file 1: Table S3. Tones with scores less than 0.5 are unlikely to be perceived in the content; scores over 0.75 mean that the measured tone will be perceived as dominant in the text [20]. Statistically significant differences (one way ANOVA and Tukey post-hoc test): “Anger”, “Fear” and “Analytical” – SA vs. PR and PLS, “Tentative” – SA vs. PLS

We used the Watson Tone Analyzer to analyze the emotional, writing and personality tone of the texts for the Scientific abstract, Plain language summary and Press release. Overall, the texts did not contain strong emotional tones except for “Sadness”, related to the use of words describing disease and suffering (Fig. 3; see the Additional file 1: Table S3 for full data set analysis), expressed as increased frequency of words indicating sickness and suffering such as “wound”, “complication”, and “infection”. “Sadness” was significantly greater in the formats targeting the press and the public (Fig. 3). The writing tone was predominantly “Analytical” for all formats, as well as “Tentative” to some degree (Fig. 3). Scientific abstracts had significantly lower analytical tone (used fewer words and phrases implying causal connections, such as “therefore” or “if...then...” etc.) than the other two formats, whereas Plain language summaries used more tentative tone than the other two formats (Fig. 3).

**Fig. 4:**
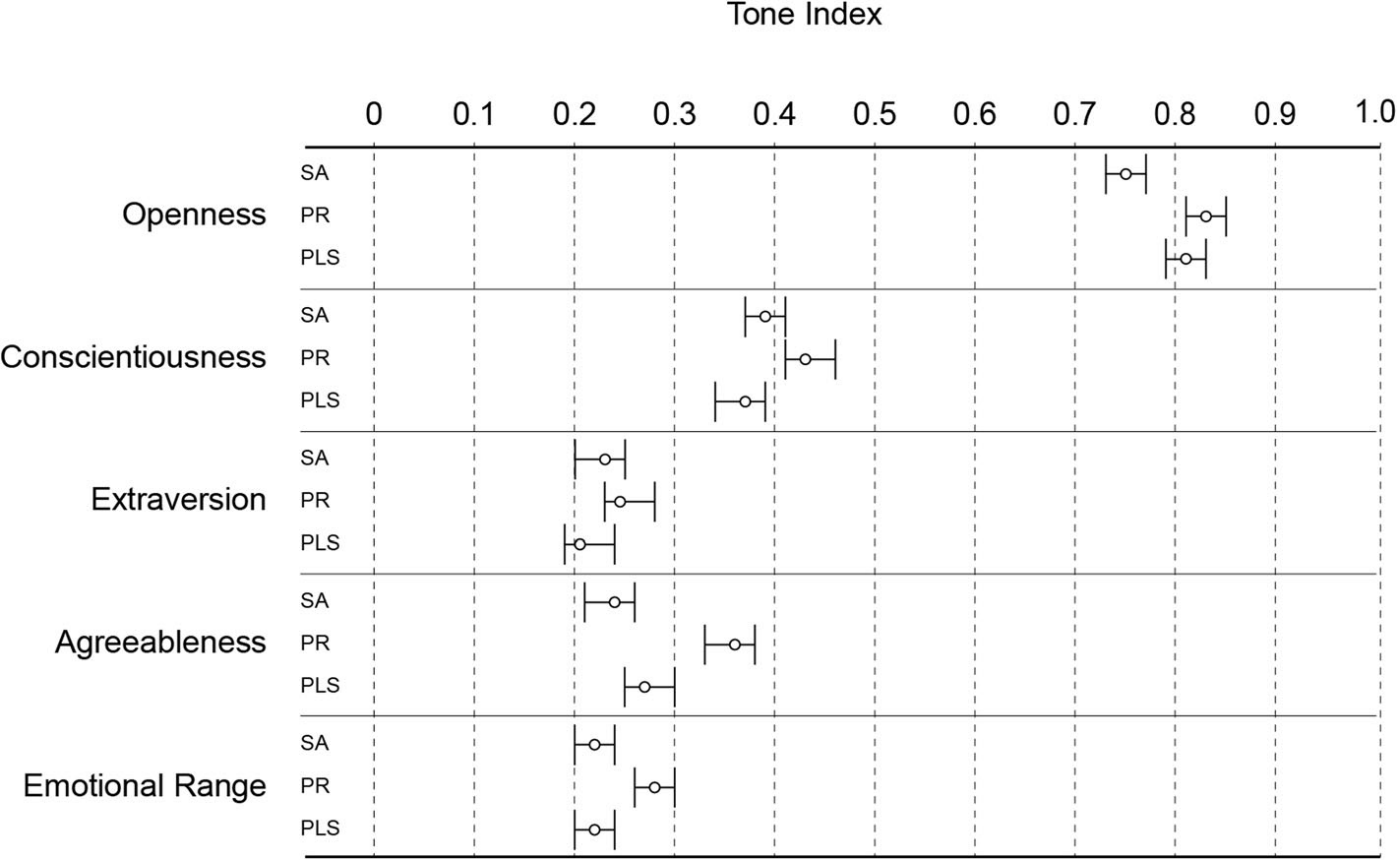
Personality tone analysis of Cochrane Scientific abstracts (SA) (*n* = 158), Press releases (PR) (*n* = 162), and English Plain language summaries (PLS) (*n* = 156). The results of IBM Watson Tone Analyzer are expressed as the probability of the output variable to be present in the text. Error bars are 95% confidence intervals. The full analysis of the dataset is available in Additional file 1: Table S3. Tones with scores less than 0.5 are unlikely to be perceived in the content; scores over 0.75 mean that the measured tone will be perceived as dominant in the text [20]. Statistically significant differences (one way ANOVA and Tukey post-hoc test): “Openness” – SA vs. PR and PLS, “Conscientiousness”, “Agreeableness” and “Emotional range” – PR vs. SA and PLS

In the analysis of the tone related to personality traits, “Openness” was the predominant psychological tone of all three formats, but was significantly lower in the Scientific abstract format than in two other formats (Fig. 4; see the Additional file 1: Table S3 for full data set analysis). The Press release format scored significantly higher than other two formats for “Conscientiousness”, “Agreeableness” and “Emotional range” (Fig. 4).

### Sentiment analysis

We used the Stanford NLP Sentiment Analysis program to estimate the sentiment in the text formats. Overall, all formats had a generally positive sentiment, but the Press release format had significantly higher positive sentiment than the other two formats (mean (95% CI) on the scale from − 1 (most negative sentiment) to + 1 (very positive sentiment): 0.09 (0.09–0.10) for the Press release format vs 0.05 (0.04–0.08) for the Scientific abstract and 0.06 (0.05–0.07) for the Plain language summary (*P* < 0.001; η^2^ = 0.07; BF_10_ = 18 × 10^5^). Compared to the other two formats, Scientific abstracts also had significantly more adjectives, nouns, cardinal numbers, dates, expressions indicating duration, numbers in general and miscellaneous entities. Plain language summaries had the least number of adjectives, proper nouns, nouns, verbs and organizations mentioned. On the other hand, Press releases mentioned significantly more people and locations compared to other formats. Details about stylistic and grammatical features of the formats are presented in the Additional file 1: Table S4.

### Linguistic inquiry and word count (LIWC) analysis

**Fig. 5:**
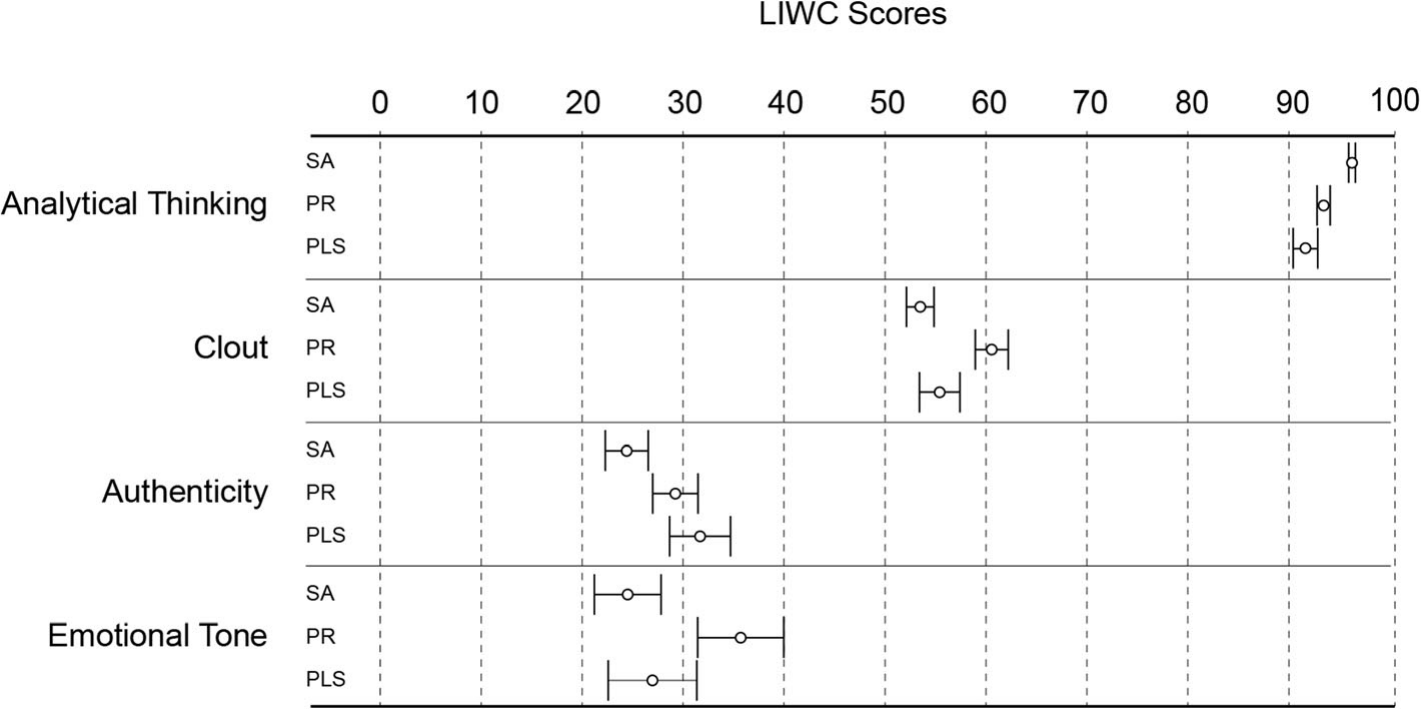
Summary variables of language style of Cochrane Scientific abstracts (SA) (*n* = 158), Press releases (PR) (*n* = 162), and English Plain language summaries (PLS) (*n* = 156) according to LIWC analyser [21]. Results represent standardized scores converted to percentiles. Error bars are 95% confidence intervals. The full analysis of the dataset is available in Additional file 1: Table S4 and S5. Statistically significant differences (one way ANOVA and Tukey post-hoc test): “Analytical thinking” – all groups different, “Authenticity” – PLS vs. SA and PR, “Clout” and “Emotional tone” – PR vs. SA and PLS

We used four summary variables from the LIWC analysis (Fig. 5). All three Cochrane summary formats had extremely high scores in the “Analytical thinking” dimension, which is characterized by words suggesting logical, formal, or hierarchical thinking. This dimension was most pronounced in the Scientific abstract format. The next predominant dimension was “Clout”, a variable that refers to confidence, leadership, or social status. According to the LIWC documentation, “a high number for Clout suggests that the author is speaking from the perspective of high expertise and is confident; low Clout numbers suggest a more tentative, humble, even anxious style” [21]. “Clout” was significantly higher in the Press release format. Scores on the “Authenticity” summary variable (language that suggests revealing oneself in an honest way) and “Emotional tone” (language suggesting either positive or negative emotion) were overall low in all formats. The Plain language summary format had significantly higher scores for “Authenticity” and the Press release format for “Emotional tone”. We also measured a number of other language dimensions, such as the use of words expressing different psychological processes, including emotional social, perceptual and cognitive processes and relativity-related words (see the Additional file 1: Table S5 for full data set analysis). The Scientific abstract format used words with less affect and positive emotions, and with less orientation on time expressions (present and future), and used the terminology related to relativity processes. The Plain language summary format had more words indicating authenticity and negative emotions. On the other hand, the Press release format used more words indicating social and perceptual processes, and words focusing on the present.

## Discussion

Our study showed low overall reading ease of textual formats that summarize the results of Cochrane systematic reviews. Although Plain language summary formats were significantly easier to read than other formats, the required literacy level was still high: on average, over 10 to 15 years of education needed for easy reading of a plain language summary across different languages, which is much higher than the recommended US 6th grade reading level [1, 2, 9].

These results have to be interpreted with the limitations inherent to all readability studies, such as appropriateness for health information [26], score variability for different languages, as was the case for the Croatian language in our study [17], and imprecise education level thresholds for brief texts, such as used in our study [27]. The reasons for differences in SMOG index for different language translations of the same information are not clear, as there is little literature on the use of the SMOG formula to compare different languages. The original SMOG formula, developed for the English language, was used for studying health-related textual information in German [28]. Further, the study that compared SMOG scores for the same text unrelated to health in English, Spanish and French found that the scores for the English were lower (i.e. the text was more readable) than in French and Spanish [29]. Our study showed the same finding for textual health information (lower SMOG score for French than for English Plain language summary), but much lower scores (i.e. better readability) for German and Croatian translations. A further limitation is the use of Cochrane as a single source of health information summaries. However, the collection of different Cochrane formats for individual systematic reviews was a suitable model for the comparison of language characteristics in different summary formats presenting the same information to different audiences. The comparison showed significant statistical differences between the formats, but no actual “clinical” difference [1, 2, 9]. We were also limited by the small sample of Cochrane press releases which were the starting point for the collection of matching text summary formats. Finally, readability can be affected by visual formatting of the text or structured presentation [30, 31], which was not tested in our study.

The low readability of different textual summaries of systematic reviews has important implications for the efforts in translating textual health evidence to different audiences. On the one hand, lower readability necessarily leads to less accessible health information, especially for patients as a non-specialist audience. On the other hand, complex scientific literature is becoming increasingly more difficult to read [32], which may decrease the accuracy of information understanding, and thus affect health decisions guided by that information [33]. This means that there should be a balance between reading ease and accurate information about health. Cochrane has been testing different interventions to this aim, including PLEACS standards (Plain Language Expectations for Authors of Cochrane Summaries) for writing plain language summaries [15]. However, the authors of Cochrane systematic reviews, who write both the Scientific abstract and the Plain language summary formats, often do not follow these standards [15]. Other formats, such as infographics, may also not be better for understanding the results of a Cochrane systematic review in comparison to a well-written plain language summary [34].

The availability of new technologies for natural (human) language processing provided us with the unique opportunity to identify and quantify affective states and subjective information in different textual systematic review summary formats. Subjective experience of people when they process a piece of information influences whether they will consider the information as truthful, whether they will like it, and whether they will have confidence in the information [11]. Linguistic characteristics of the text contribute to the way people react to health information [35]. Sentiment analysis of physician-patient communication showed that patients adhered more to the advice of physicians who used fewer words related to negative emotion and fewer singular first-person pronouns [36]. Clinical advertisements by cancer centers in US consumer magazines and TV networks were shown to use emotional appeals to evoke hope and fear, but not providing sufficient information about objective information about risks vs. benefits, and costs [37].

We used three different natural language analysis tools and assessed different language traits to compare textual formats about health evidence targeting different audiences. Scientific abstracts had not only the longest format but also fewer emotional words. Scientific abstracts are intended for professionals and emotional “coldness” is to be expected as it presents information needed for physicians and other experts to gain greater insight into the methodological aspect of research required for critical assessment [38]. In contrast to this, Press releases were written more engagingly, had more clout (high expertise and confidence), as well as an overall more positive sentiment, more positive and engaging personality traits and were more engaging than either Scientific abstracts or Plain language summaries. Plain language summaries also had the highest “Tentative” tone, which may be related to difficulties in explaining complex scientific findings, which are by nature contradictory and in some way uncertain, and tentative [39].

As evidenced by data from all three language analysis tools, the style of writing in Press releases followed the standards of the health journalistic profession to “get attention, arouse interest and stir emotions” [38]. On the other hand, the language style and emotional content of the Plain language summaries was more similar to that of the Scientific abstracts, which can be expected from texts written by the same person – authors of the systematic review. This left the Plain language summaries in a metacognitive “no man’s land”: they lacked the cognitive fluency to facilitate judgment about the presented information but also lacked the details for a full critical interpretation of the methods, findings and limitations of the research. We also found apparently contradictory finding by the two different language analysis tools, like lower analytical tone for the Scientific abstracts, as measured by Watson Tone Analyzer (Fig. 3) but higher analytical thinking according to LIWC analyzer (Fig. 5). This apparent difference may be related to the different dictionaries used by the two methods, but it is not possible to precisely identify its source as the dictionaries for the two tools are not openly accessible.

It is difficult to assess the “clinical” significance of the statistically significant differences observed for language characteristics in our study, as there are, to the best of our knowledge, no similar linguistic comparison studies of different textual formats for the same health information. Future research should test hypotheses that can be generated from our study, such as how linguistic characteristics of the text affect the ability of a reader to understand and interpret the presented information.

General health literacy research ranges between two extremes: the proposition that news stories should read like research articles in order to accurately express scientific findings [40], and the proposition that news stories should involve a more human, emotional element into the text in order to improve understanding of health information [41, 42]. Our study showed that the Press releases about Cochrane systematic reviews followed the latter approach of more conversational language and paring of the scientific details, although they were aimed at supposedly highly health-literate consumers – health journalists [38]. Plain language summaries had a mix of formats both for high and low health-literate readers, which may not be an efficient method of health evidence translation to the patients and lay public in general [1, 2, 9].

## Conclusion

Our study found that the language of professionally written press releases was generally more emotionally and cognitively engaging than that of scientific abstracts and plain language summaries, which are written by the authors of systematic reviews. Future research using novel language processing tools may help bring together the needs and wishes of different health information users and provide more evidence on the text characteristics that have impact on the cognitive processing of the users, as well as on the importance of presenting sufficient details about scientific validity and interpretation of health evidence.

## Additional file

Additional file 1: Full linguistic analysis, Tables S1-S5. (DOCX 28 kb)

## Abbreviations

ANOVA: Analysis of variance
BF: Bayes Factor
CI: Confidence interval
LIWC: Linguistic Inquiry and Word Count
NLP: Natural language processing
PLEACS: Plain Language Expectations for Authors of Cochrane Summaries
PLS: Plain language summary
SMOG: Simple Measure Of Gobbledygook
