# Supplementary material for: Languages for different health information readers: multitrait-multimethod content analysis of Cochrane systematic reviews textual summary formats
Source: BMC Med Res Methodol. 2019 Apr 5;19:75. doi: 10.1186/s12874-019-0716-x (PMC6451281; doi:10.1186/s12874-019-0716-x)
Supplement: Supplementary file 1 — Full linguistic analysis, Tables S1-S5. (DOCX 28 kb) [file 12874_2019_716_MOESM1_ESM.docx]

Karačić, Dondio et al. Languages for different health information readers: Multitrait-multimethod content analysis of systematic reviews textual summary formats

**Supplement –** Full data set linguistic analysis, eTables 1-5

**eTable 1. Readability characteristics (median, 95% confidence interval) of different summary information formats for Cochrane systematic reviews**

**eTable 2. Readability characteristics (median, 95% confidence interval) of different translations of plain language summaries**

**eTable 3. Tone analysis using IBM Watson Tone Analyzer analysis for Cochrane scientific abstracts (SA, n=158), press releases (PR, n=162) and plain language summaries (PLS, n=156)**

**eTable 4. Sentiment analysis using Stanford NLP program for Cochrane scientific abstracts (SA, n=158), press releases (PR, n=162) and plain language summaries (PLS, n=156)**

**eTable 5. LIWC language analysis for Cochrane scientific abstracts (SA, n=158), press releases (PR, n=162) and plain language summaries (PLS, n=156)**

**eTable 1. Readability characteristics (median, 95% confidence interval) of different summary information formats for Cochrane systematic reviews**

| **Output variables** | **A – Press releases (n=162)** | **B – Cochrane Clinical Answers (n=35)** | **C – Scientific abstract (n=158)** | **D – Plain language summary (n=156)** | **P*** |
| --- | --- | --- | --- | --- | --- |
| Word count | 407.5 (388.1-425.9)^BCD^ | 178 (143.0-208.5)^ACD^ | 555.5 (495.6-600.8)^ABD^ | 334.5 (286.0-363.4)^ABC^ | P<0.001 |
| Sentence count | 17.0 (16.0-18.0)^BC^ | 8.0 (7.0-10.0)^ACD^ | 26.0 (24.0-28.0)^ABD^ | 17.0 (15.0-19.0)^BC^ | P<0.001 |
| Words per sentence | 23.6 (23.1-24.0)^BCD^ | 20.4 (15.8-21.1)^AC^ | 20.8 (20.3-21.4)^ABD^ | 18.3 (17.7-19.1)^AC^ | P<0.001 |
| Syllables per word | 1.8 (1.8-1.8)^BCD^ | 2 (1.9-2.0)^ACD^ | 1.9 (1.9-1.9)^ABD^ | 1.8 (1.8-1.8)^ABC^ | P<0.001 |
| SMOG index | 15.4 (15.1-15.5)^CD^ | 15.2 (14.8-16.6)^D^ | 15.6 (15.3-15.9)^AD^ | 14.7 (14.4-15.0)^ABC^ | P<0.001 |

*Kruskal Wallis non-parametric test and *post-hoc* Conover Iman test. Superscript letter indicates significant difference from relevant study groups designated with the same letter.

**eTable 2. Readability characteristics (median, 95% confidence interval) of different translations of plain language summaries**

| **Output variables** | | **A – English (n=156)** | | **B – German (n=41)** | **C– French (n=101)** | **D – Croatian (n=156)†** | **P** |
| --- | --- | --- | --- | --- | --- | --- | --- |
| Word count | 334.5 (286.0-363.4)^B^ | | 378 (353.4-411.1)^AD^ | | 354 (297.1-396.7) | 335 (287.3-355.0)^B^ | P<0.001 |
| Sentence count | 17.0 (15.0-19.0)^B^ | | 22.0 (19.5-25.0)^ACD^ | | 15.0 (13.0-17.0)^B^ | 17.0 (15.0-19.0)^B^ | P=0.007 |
| Words per sentence | 18.3 (17.7-19.1)^C^ | | 17.9 (16.9-18.7)^C^ | | 21.7 (20.9-22.7)^ABD^ | 18.1 (17.5-19.0)^C^ | P<0.001 |
| Syllables per word | 1.8 (1.8-1.8)^BCD^ | | 2.1 (2.1-2.2)^ACD^ | | 1.8 (1.8-1.8)^ABD^ | 2.5 (2.5-2.5)^ABC^ | P<0.001 |
| SMOG index | 14.7 (14.4-15.0)^BCD^ | | 12.0 (11.7-12.3)^ACD^ | | 15.3 (14.8-15.5)^ABD^ | 10.3 (9.9-10.3)^ABC^ | P<0.001 |

*Kruskal Wallis non-parametric test and *post-hoc* Conover Iman test. Superscript letter(s) indicates significant difference from relevant study groups designated with the same letter.

†SMOG index for Croatian language was calculated according to the formula adapted to the Croatian language (Brangan S. Development of SMOG-Cro readability formula for healthcare communication and patient education. Coll Antropol. 2015;39(1):11-20.).

**eTable 3. Tone analysis using IBM Watson Tone Analyzer analysis for Cochrane scientific abstracts (SA, n=158), press releases (PR, n=162) and plain language summaries (PLS, n=156)**

| **Output variable*** | **Format** | **Mean (95% confidence interval)** | **Statistics†** |
| --- | --- | --- | --- |
| **Emotion variables:** |  |  |  |
| Anger | SA | 0.22 (0.21-0.24) | SA vs. PR and PLS: |
|  | PR | 0.12 (0.11-0.14) | P<0.001; η^2^=0.12; BF_10_=1.29×10^11^ |
|  | PLS | 0.15 (0.13-0.17) |  |
| Fear | SA | 0.22 (0.19-0.24) | SA vs. PR and PLS: |
|  | PR | 0.32 (0.29-0.35) | P<0.001; η^2^=0.06; BF_10_=8.65×10^3^ |
|  | PLS | 0.29 (0.27-0.32) |  |
| Joy | SA | 0.26 (0.23-0.29) |  |
|  | PR | 0.30 (0.27-0.33) | P=0.109; BF_10_=0.191 |
|  | PLS | 0.27 (0.24-0.30) |  |
| Sadness | SA | 0.50 (0.48-0.52) | SA vs. PR and PLS: |
|  | PR | 0.55 (0.53-0.57) | P<0.001; η^2^=0.03; BF_10_=18.07 |
|  | PLS | 0.54 (0.52-0.56) |  |
| **Writing variables:** |  |  |  |
| Analytical | SA | 0.81 (0.79-0.82) | SA vs. PR and PLS: |
|  | PR | 0.86 (0.84-0.87) | P<0.001; η^2^=0.05; BF_10_=3.67×10^3^ |
|  | PLS | 0.83 (0.82-0.85) |  |
| Tentative | SA | 0.50 (0.50-0.57) | SA vs. PLS: |
|  | PR | 0.57 (0.53-0.61) | P=0.005, η^2^=0.02; BF_10_=3.23 |
|  | PLS | 0.62 (0.59-0.66) |  |
| **Personality variables:‡** |  |  |  |
| Openness | SA | 0.75 (0.73-0.77) | SA vs. PR and PLS: |
|  | PR | 0.83 (0.81-0.85) | P<0.001; η^2^=0.07; BF_10_=5.47×10^4^ |
|  | PLS | 0.81 (0.79-0.83) |  |
| Conscientiousness | SA | 0.39 (0.37-0.41) | PR vs. SA and PLS |
|  | P | 0.43 (0.41-0.46) | P<0.001; η^2^=0.04; BF_10_=168.53 |
|  | PLS | 0.37 (0.34-0.39) |  |
| Extraversion | SA | 0.23 (0.20-0.25) |  |
|  | PR | 0.25 (0.23-0.28) | P=0.038; BF_10_=0.518 |
|  | PLS | 0.21 (0.19-0.24) |  |
| Agreeableness | SA | 0.24 (0.21-0.26) | PR vs. SA and PLS: |
|  | PR | 0.36 (0.33-0.38) | P<0.001; η^2^=0.09; BF_10_=2.52×10^7^ |
|  | PLS | 0.27 (0.25-0.30) |  |
| Emotional range | SA | 0.22 (0.20-0.24) | PR vs. SA and PLS: |
|  | PR | 0.28 (0.26-0.30) | P<0.001; η^2^=0.04; BF_10_=283.86 |
|  | PLS | 0.22 (0.20-0.24) |  |

*The results are expressed as the probability of the output variable to be present in the text. Tones with scores less than 0.5 are not presented (available in Supplementary documentation) as they are unlikely to be perceived in the content; scores over 0.75 mean that the measured tone will be perceived in this text (IBM Cloud Docs. Tone Analyzer; <https://console.bluemix.net/docs/services/tone-analyzer/using-tone.html#using-the-general-purpose-endpoint>).

**†**One way ANOVA and Tukey post-hoc test; η^2^ – effect size measure; BF_10_ – Bayes factor for testing the probability that alternative hypothesis is more likely compared to null hypothesis.

‡These output variables are related to five personality traits .

**eTable 4. Sentiment analysis using Stanford NLP program for Cochrane scientific abstracts (SA, n=158), press releases (PR, n=162) and plain language summaries (PLS, n=156)**

| **Output variable** | **Format** | **Mean (95% confidence interval)** | **Statistics*** |
| --- | --- | --- | --- |
| Overall sentiment | SA | 0.05 (0.04-0.08) | PR vs. SA and PLS |
|  | PR | 0.09 (0.08-0.10) | P<0.001; η^2^=0.07; BF_10_=18x10^5^ |
|  | PLS | 0.06 (0.05-0.07) |  |
| Maximum depth among all the sentences in the document† | SA | 19.95 (19.40-20.50) |  |
|  | PR | 20.93 (20.39-21.48) | P=0.022; BF_10_=0.854 |
|  | PLS | 20.03 (19.48-20.58) |  |
| Average depth of the sentences in the document† | SA | 11.30 (11.11-11.49) | All groups different: |
|  | PR | 12.87 (12.68-13.06) | P<0.001; η^2^=0.22; BF_10_=9.70×10^22^ |
|  | PLS | 12.19 (12.00-12.38) |  |
| Number of superlative adjectives | SA | 0.14 (0.11-0.17) |  |
|  | PR | 0.20 (0.16-0.24) | P=0.130; BF_10_=0.161 |
|  | PLS | 0.17 (0.13-0.22) |  |
| Number of comparative adjectives | SA | 0.47 (0.400.54) | SA vs. PR and PLS |
|  | PR | 0.72 (0.65-0.80) | P=<0.001; η^2^=0.04; BF_10_=115.71 |
|  | PLS | 0.65 (0.54-0.75) |  |
| Number of adjectives | SA | 9.14 (8.83-9.45) | PR vs. SA and PLS |
|  | PR | 8.21 (7.93-8.49) | P<0.001; η^2^=0.04; BF_10_=66.71 |
|  | PLS | 9.07 (8.63-9.52) |  |
| Number of proper nouns | SA | 2.67 (2.45-2.89) | All groups different |
|  | PR | 4.01 (3.78-4.23) | P<0.001; η^2^=0.48; BF_10_=1.59×10^64^ |
|  | PLS | 0.87 (0.69 - 1.05) |  |
| Number of nouns | SA | 29.14 (28.79-29.49) | All groups different: |
|  | PR | 24.90 (24.53-25.28) | P<0.001; η^2^=0.32; BF_10_=1.03×10^36^ |
|  | PLS | 29.09 (27.58-28.62) |  |
| Number of verbs | SA | 10.47 (10.18-10.76) | All groups different: |
|  | PR | 15.07 (14.78-15.35) | P<0.001; η^2^=0.45; BF_10_=1.22×10^58^ |
|  | PLS | 14.25 (13.80-14.71) |  |
| Number of cardinal numbers in the document | SA | 6.63 (6.22-7.05) | All groups different: |
|  | PR | 1.78 (1.62-1.85) | P<0.001; η^2^=0.55; BF_10_=2.57×10^78^ |
|  | PLS | 2.64 (2.35-2.94) |  |
| Number of persons mentioned in the document | SA | 0.07 (0.04-0.10) | PR vs. SA and PLS: |
|  | PR | 0.89 (0.84-0.95) | P<0.001; η^2^=0.67; BF_10_=2.56×10^118^ |
|  | PLS | 0.06 (0.03-0.10) |  |
| Number of dates mentioned in the document | SA | 1.36 (1.20-1.52) | SA vs. PR and PLS: |
|  | PR | 0.56 (0.47-0.64) | P<0.001; η^2^=0.21; BF_10_=1.77×10^21^ |
|  | PLS | 0.53 (0.43-0.63) |  |
| Number of locations mentioned in the document | SA | 0.15 (0.10-0.19) | PR vs. SA and PLS: |
|  | PR | 0.68 (0.60-0.75) | P<0.001; η^2^=0.27; BF10=6.54×10^29^ |
|  | PLS | 0.14 (0.07-0.21) |  |
| Number of organizations mentioned in the document | SA | 1.53 (1.37-1.70) | All groups different: |
|  | PR | 2.35 (2.17-2.54) | P<0.001; η^2^=0.40; BF_10_=1.74×10^49^ |
|  | PLS | 0.32 (0.20-0.45) |  |
| Number of "numbers" in the document | SA | 4.43 (4.11-4.75) | SA vs. PR and PLS: |
|  | PR | 1.30 (1.20-1.40) | P<0.001; η^2^=0.48; BF_10_=4.65×10^63^ |
|  | PLS | 1.85 (1.65-2.06) |  |
| Number of expressions indicating "duration" | SA | 0.96 (0.79-1.21) |  |
|  | PR | 0.79 (0.65-0.93) | P=0.184; BF_10_=0.116 |
|  | PLS | 0.99 (0.80-1.19) |  |
| Number of "miscellaneous entities" in the document‡ | SA | 0.31 (0.24-0.37) | SA vs. PR and PLS: |
|  | PR | 0.19 (0.13-0.25) | P<0.001; η^2^=0.03; BF_10_= 49.37 |
|  | PLS | 0.13 (0.08-0.19) |  |

*One way ANOVA and Tukey post-hoc test; η^2^ – effect size measure; BF_10_ – Bayes factor for testing the probability that alternative hypothesis is more likely compared to null hypothesis.

†Average and Maximum depth relate to the depth of the parsing tree for each sentence. A parsing tree is an ordered, rooted tree (graph) that represents the syntactic structure of a sentence according to the English grammar. The deeper the tree, the more complex the sentence (Bird S, Klein E, Loper E. 8. Analyzing sentence structure. In: Bird S, Klein E, Loper E. Natural language processing with python – analyzing text with the natural language toolkit. Sebastopol, CA, USA: O'Reilly Media, 2009. Available from: <http://zempirians.com/ebooks/Steven%20Bird,%20Ewan%20Klein,%20Edward%20Loper-Natural%20Language%20Processing%20with%20Python-O'Reilly%20Media%20(2009).pdf>).

‡All remaining entities that are not dates, persons, number, locations, organizations.

**eTable 5. LIWC language analysis for Cochrane scientific abstracts (SA, n=158), press releases (PR, n=162) and plain language summaries (PLS, n=156)**

| **Output variable*** | **Format** | **Mean (95% confidence interval** | **Statistics†** |
| --- | --- | --- | --- |
| **Summary variables:** |  |  |  |
| Analytical thinking | SA | 96.21 (95.89-96.54) | All groups different: |
|  | PR | 93.40 (92.75-94.04) | P<0.001; η^2^=0.12; BF_10_=7.57×10^10^ |
|  | PLS | 91.60 (90.39-92.81) |  |
| Clout | SA | 53.46 (52.09-54.83) | PR vs. SA and PLS: |
|  | PR | 60.53 (58.90-62.16) | P<0.001; η^2^=0.07; BF_10_=53.99×10^5^ |
|  | PLS | 55.38 (53.38-57.38) |  |
| Authenticity | SA | 24.39 (22.27-26.52) | PLS vs. SA and PR: |
|  | PR | 29.21 (26.97-31.45) | P<0.001; η^2^=0.04; BF_10_=69.33 |
|  | PLS | 31.66 (28.64-34.67) |  |
| Emotional tone | SA | 24.49 (21.18-27.80) | PR vs. SA and PLS: |
|  | PR | 35.68 (31.42-39.94) | P<0.001; η^2^=0.04; BF_10_=61.27 |
|  | PLS | 26.94 (22.56-31.32) |  |
| **Individual variables:** |  |  |  |
| Affect (Total) | SA | 3.99 (3.75-4.22) | SA vs. PR and PLS: |
|  | PR | 4.70 (4.41-4.99) | P<0.001; η^2^=0.05; BF_10_=6.17×10^3^ |
|  | PLS | 5.04 (4.71-5.37) |  |
| Positive emotion | SA | 1.75 (1.58-1.92) | SA vs. PR and PLS: |
|  | PR | 2.51 (2.28-2.73) | P<0.001; η^2^=0.05; BF_10_=1.36×10^3^ |
|  | PLS | 2.29 (2.02-2.57) |  |
| Negative emotion | SA | 2.20 (2.03-2.38) | PLS vs. SA and PR: |
|  | PR | 2.16 (1.95-2.37) | P=0.001; η^2^=0.03; BF_10_=19.91 |
|  | PLS | 2.65 (2.42-2.89) |  |
| Social | SA | 4.20 (3.95-4.45) | All groups different |
|  | PR | 6.43 (6.07-6.78) | P<0.001; η^2^=0.14; BF_10_=9.75×10^12^ |
|  | PLS | 5.60 (5.15-6.05) |  |
| Cognitive Processes | SA | 11.84 (11.48-12.20) | SA vs. PR and PLS: |
|  | PR | 12.73 (12.32-13.14) | P=0.001; η^2^=0.04; BF_10_=140.49 |
|  | PLS | 13.12 (12.63-13.60) |  |
| Perceptual processes | SA | 1.13 (0.96-1.29) | SA vs. PR and PLS: |
|  | PR | 2.18 (1.92-2.44) | P<0.001; η^2^=0.07; BF_10_=1.67×10^6^ |
|  | PLS | 1.68 (1.42-1.95) |  |
| Time – Past focus | SA | 3.47 (3.29-3.64) |  |
|  | PR | 3.64 (3.46-3.83) | P=0.351; BF_10_=0.063 |
|  | PLS | 3.66 (3.42-3.90) |  |
| Time – Present focus | SA | 2.93 (2.76-3.09) | All groups different: |
|  | PR | 6.33 (6.07-6.60) | P<0.001; η^2^=0.45; BF_10_=2.40×10^57^ |
|  | PLS | 5.27 (4.97-5.57) |  |
| Time – Future focus | SA | 0.34 (0.29-0.39) | SA vs. PR and PLS: |
|  | PR | 0.69 (0.62-0.77) | P<0.001; η^2^=0.09; BF_10_=4.92 |
|  | PLS | 0.64 (0.54-0.74) |  |
| Relativity | SA | 10.15 (9.77-10.53) | SA vs. PR and PLS: |
|  | PR | 12.01 (11.60-12.42) | P<0.001; η^2^=0.07; BF_10_=6.63×10^5^ |
|  | PLS | 11.56 (11.03-12.09) |  |

*Summary variables represent standardized scores converted to percentiles. Scores for all other variables represent percentage of words from LIWC dictionary that define that individual variable (LIWC2015_LanguageManual; <https://repositories.lib.utexas.edu/bitstream/handle/2152/31333/LIWC2015_LanguageManual.pdf>).

**†**One way ANOVA and Tukey post-hoc test; η2 – effect size measure; BF10 – Bayes factor for testing the probability that alternative hypothesis is more likely compared to null hypothesis.
